# Parents’ Perspectives of an Arts Engagement Program Supporting Children with Anxiety

**DOI:** 10.3390/ijerph20186771

**Published:** 2023-09-16

**Authors:** Diane Macdonald, Jin Han, Emma Elder, Katherine M. Boydell

**Affiliations:** 1 Black Dog Institute, Sydney 2031, Australia; hjin34974@gmail.com (J.H.); emma.elder@blackdog.org.au (E.E.); k.boydell@blackdog.org.au (K.M.B.); 2 Faculty of Health & Medicine, University of New South Wales, Sydney 2034, Australia; 3 Dalla Lana School of Public Health, Faculty of Medicine, University of Toronto, Toronto, ON M5G 1V7, Canada

**Keywords:** child anxiety, mental health, arts engagement programs, parents and children, community care, galleries and wellbeing, anxiety, arts-based mental health program

## Abstract

Arts engagement programs (AEPs) are non-clinical, structured programs led by artists and educators to support mental health and wellbeing. While evidence demonstrates positive mental health outcomes in adult AEPs, studies of childhood AEPs remain sparse. We created a gallery-based AEP (Culture Dose for Kids) for children with anxiety based on a successful arts engagement pilot for adults with depression. We questioned whether our tailored-for-children adult program would effectively and feasibly support children’s mental health. Through parents’ perspectives and feedback, this study tested the program’s acceptability, feasibility, and effectiveness with children with anxiety. Quantitative and qualitative measures were used to determine whether the program was an effective and acceptable mental health support for children with anxiety. Our findings revealed that the program positively and significantly impacted parental perceptions of their child’s anxiety. Our findings illustrate depictions of improved mood, confidence, and sense of empowerment in the child, qualities associated with resilience and mental wellbeing. Open-ended activities provided opportunities for connection, creativity, and experimentation—sources of strength for improving mental health. This study adds to the small but growing evidence base supporting the role of arts-based community care in youth mental health and wellbeing.

## 1. Introduction

Late childhood and early adolescence (about ages 9–12) are times of heightened emotional turbulence. Transitioning from childhood to adolescence presents many physical, intellectual, social developmental, and personality changes. This period can also see the onset of mental health issues, and one of the most common issues for young people is anxiety disorder [1].

In developed countries, an estimated nine per cent of young people experience anxiety disorders, and this is likely an underestimate [2]. Although some degree of anxiety and fear is normal and healthy for children, high levels of anxiety can cause considerable harm and distress to children and their families [3]. High and chronic levels of anxiety include excessive fear and related behavioural disturbances like avoidance behaviours or repeated actions to prevent a feared situation from occurring [3]. Anxiety can affect, among other things, the child’s physical health (headaches, stomach aches, trouble sleeping) and emotional health (fears of being laughed at or afraid to take part in events) [4]. Anxiety can also limit essential socialisation and increase feelings of isolation, affecting children, parents, and family.

If left unresolved, mental health disorders in childhood can have an enduring negative impact. Helping children in early adolescence may present an optimal opportunity to support and help young people with their mental health needs. Indeed, the long-term benefits are magnified when children receive treatment around the onset of an anxiety disorder [5].

Despite recent investments in youth mental health in Australia, young individuals typically have low engagement with mental health services. Estimates suggest that only about a third of young individuals with a mental disorder have received formal mental health care [6]. When care is accessed, it is often found to be inappropriate for them [5]. While youth mental health care has improved over the last few decades, systemic weaknesses in youth mental healthcare have resulted in missed opportunities for prevention and early help [7]. Beyond systemic weaknesses, long-held cultural attitudes about and stigma around mental illness and seeking help for mental illness create additional barriers to help [8]. The knock-on effect of stigma in mental illness effectively limits access to treatment with lifelong consequences for children [9]. Thus, the need for accessible, trusted, and non-stigmatising mental health interventions and support for children and early adolescents is urgent and vital.

Parents can play an essential role in the development, prolongation, and treatment outcomes of anxiety disorders in children [10]. However, for clarity and brevity of space, this paper does not consider the parents’ roles in contributing to or explaining their child’s anxiety, or otherwise. Rather, we focus on the parent’s perspectives of an arts-based intervention that aims to support their child’s wellbeing, which we describe below.

### 1.1. Arts Engagement and Mental Health

In the context of our research, arts engagement programs (AEPs) refer to non-clinical, structured programs led by artists and educators to support wellbeing [11]. AEPs have been found to reduce stress and anxiety, as well as increase confidence and self-esteem in adults [12,13]. AEPs take a broader approach to health care, incorporating aspects of mental health, including social inclusion, creativity, and enjoyment [14,15]. Evidence demonstrates positive outcomes in adults as a result of AEP interventions, including facilitating social inclusion, reducing anxiety and depression symptoms, increasing self-confidence, producing feelings of empowerment and wellbeing, and enhancing quality of life [11,12].

AEPs acknowledge that access to cultural and artistic pursuits can enhance health outcomes by fostering positive psychological, social, and behavioural responses [11,12,13]. These programs play a pivotal role in supporting mental health as they serve as a platform for self-expression, enabling individuals to convey their emotions, thoughts, and experiences in ways that may be difficult to express verbally. This connection to self, in turn, deepens an understanding of one’s emotions, nurturing wellbeing. Furthermore, engagement in arts-related activities, such as painting, drawing, dance, or music, can aid relaxation, diminish stress, and improve mood [14,15]. Participating in creative endeavours often requires focused concentration and immersion, facilitating a state of mindfulness. AEPs offer respite from daily routines to counteract negative thought patterns. They also contribute to a sense of achievement and bolster self-esteem and social connections, fostering a sense of belonging [11,12,13,14,15].

A study of an arts–mental health relationship found that adults who took part in two or more hours per week of arts engagement reported significantly better mental wellbeing than other levels of engagement [12]. Evidence demonstrates positive outcomes in adults as a result of these interventions, including facilitating social inclusion and belonging, reducing anxiety and depression symptoms, increasing self-confidence, producing feelings of empowerment and wellbeing, and enhancing quality of life [12]. Among the range of community options available, arts interventions are well suited to address the needs of young people.

Arts engagement for better mental health in young people encompasses a variety of genres, from circus [16] and creative writing [17] to visual art programs (our specific interest) [18,19], among others. However, the peer-reviewed evidence is sparse on the role of AEPs in youth mental health worldwide, a gap reduced by our research [20].

Among the few published articles including parents and children together in arts engagement programs to support children’s mental health, we identified few studies. In a remote Aboriginal community, Stock, Mares, and Robinson [21] found that a parent/child art and storytelling program engaged and connected parents and children, providing a platform for parents to reflect on various aspects of their own and their children’s journeys. Furthermore, Mak and Fancourt [19] determined that the positive impact of AEPs may be magnified if parents are included with the children. This study aims to add to these findings.

### 1.2. Culture Dose for Kids

Culture Dose for Kids (“Culture Dose” is a play on words that combines two distinct concepts to create a catchy title for the program. “Culture” encompasses artful experiences. “Dose” refers to the way medicine is administered to achieve a certain effect. Thus, “Culture Dose” suggests the idea that experiencing cultural activities like arts engagement in a deliberate and controlled manner can have a positive impact on an individual’s wellbeing, similar to how a medicine dose can improve health) (CDK) is an AEP for young people experiencing anxiety and their parents/caregivers (from now on, parents). CDK was developed through collaboration between mental health researchers and art educators from the Art Gallery of New South Wales (Sydney) to support children with anxiety through weekly engagement with the arts in a gallery setting. Expert and experienced art educators guide and help young people connect to their feelings, thoughts, and imagination to build self-confidence and resilience. CDK is based upon a successful arts engagement pilot for adults with depression that showed a decrease in anxiety and depression and increased social inclusion [11]. CDK considers the child and parent in people–place–activity–time interactions to build upon community and family strengths. This paper examines the 2022 CDK pilot as a trial for use in a more extensive research program.

### 1.3. CDK Program

CDK took place over eight Saturdays in May and June 2022. The sessions included four segments: 45 min of guided, meditative, slow-looking (viewing an artwork for an extended time and discussing questions about the artwork as a group) at three thematic artworks (parents and children were in separate parallel sessions); morning tea; an hour of playful open-ended art creation together (inspired by the artworks they had just engaged with); and closing discussions and sharing art creations. Thematic content for each session was identified using an Australian longitudinal study that captured young people’s worries and concerns together with lived experience consultations [22]. Our published protocol paper provides a more detailed accompaniment to this section [23].

### 1.4. Research Aim

**Figure 1:**
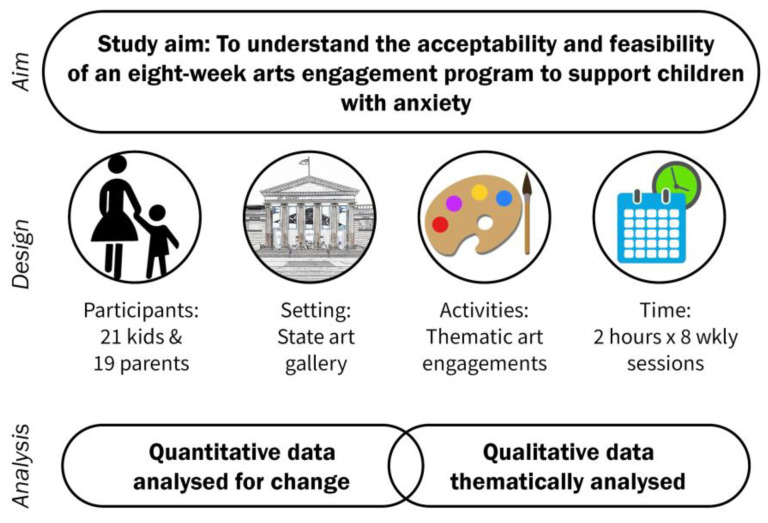
Culture Dose for Kids research design.

In this study, we sought to understand the parental perspectives (acceptability), feasibility (viability and practicality), and effectiveness of an eight-week AEP pilot at a state gallery for children with anxiety. The goal was addressed through qualitative (interviews, observations, and evaluations) and quantitative (anxiety scale and evaluative) measures. This program considered a people (parents and children)–place (gallery setting)–activity (the arts)–time (eight weeks) approach, as shown in Figure 1.

## 2. Materials and Methods

Our study design was informed by consultation with (a) parents with lived and living experience of their child’s anxiety; (b) in-house mental health experts in lived experience engagement; (c) child and adolescent mental health experts; and (d) the Art Gallery’s decades of experience running similar programs for diverse populations. This project was reviewed and approved by the University of New South Wales’ Human Research Ethics Committee (approval number: HC211020). Participant involvement in the study was voluntary. A study information sheet and consent form were provided at the outset of the research and informed consent was gained from participants prior to their participation.

### 2.1. Recruitment and Participants

After receiving ethical approval, recruitment occurred through social media in March 2022. After receiving 30 applicants who stated that their child was (1) experiencing mild anxiety (as identified by the parent); (2) in primary school, aged 9–12; (3) able to function in a group environment; and (4) able to take part in research, we closed the application process. All 30 applicants met the criteria and were accepted.

### 2.2. Participant Sample

Nineteen families from the Sydney metropolitan region attended and participated in the eight-week CDK research study, including 21 children (10 girls/11 boys) (22 children attended some sessions; however, 1 child withdrew after four weeks and 4 parents completed RCADS-P25 for the first session only). Two families had two children (each) participate. The mean number of sessions attended by children was six (a COVID-19 outbreak affected several sessions). Participants were not reimbursed for their participation.

### 2.3. Data Collection

This project used quantitative and qualitative research methodologies to collect data presented in Table 1. After consultation with in-house experts, our quantitative measures included evaluation ratings and the Revised Child Anxiety and Depression Scale Parent version (RCADS-P25), which assessed the child’s anxiety and depressive symptoms from the parent’s perspective [24]. RCADS are efficient and reliable measuring tools designed for repeated use to evaluate anxiety and depressive problems in children over time [25]. RCADS-P25 is a widely used parent-reported questionnaire designed to assess symptoms of anxiety and depression in children and adolescents. Its factor structure consists of 25 Likert-type questions corresponding to a specific type of anxiety or depressive symptom [25]. The scale’s reliability is evaluated through internal consistency and test–retest stability, both of which contribute to its validity as a measure of anxiety and depression symptoms in children [24,25]. Although the RCADS-P tool measures depression levels, we were not focused on depression in this study and have not included this construct in our paper.

RCADS were offered to parents on three occasions: at the first and last sessions, with a follow-up administered three months later by email. RCADS were employed to measure change over time, not to diagnose any disorder. We report on the anxiety component only in this study.

In addition, we collected quantitative data from parents through anonymous evaluation forms, asking questions about the program’s acceptability. Questions centred around their experiences of CDK and its impact on their wellbeing and social connectedness and whether they would recommend CDK to other parents with children with anxiety as an acceptable intervention.

Qualitative enquiry can enhance findings by encompassing and illustrating the complexity of human health and social behaviours [26,27]. Qualitative methods included interview questions (Supplementary Materials File SI) and evaluation questions (Supplementary Materials File S2). On the advice of the parents, the children were not interviewed. The parents believed one-on-one interviews would be too confrontational for the children. In addition, the first author recorded her observations immediately following each session, focusing on context, processes, and interactions.

### 2.4. Analysis

Four data sources were analysed: interview transcripts, observational notes, written anonymous evaluations, and RCADS-P25 scores.

#### 2.4.1. Quantitative

RCADS data, including children’s gender, number of sessions completed, and RCADS-P25 scores, were exported to SPSS Version 26 for analysis. Changes in anxiety as reported by parents met the assumption of normal distribution (*p* > 0.05 for Shapiro–Wilk [28] tests), meaning the data were spread out evenly and no major outliers were found. Missing values were checked and found to be random and not related to gender, the number of sessions completed, or changes in anxiety, indicating the missing values did not bias our results. A complete case analysis was adopted due to the nature of the pilot study and the missing data were assumed to be missing completely at random (MCAR). Descriptive analysis, including mean (M) and standard deviation (SD), was used to describe symptom changes across the time points. A paired samples t-test was used to analyse the changes from baseline to post-intervention in the RCADS P-25 scores. The follow-up time (three months after) data were removed from the analysis due to over 50% missing data (45.4% completion rate). Significance levels were set at *p* < 0.05, meaning that a *p*-value of <0.05 considers the results statistically significant, suggesting that the changes were unlikely to occur by chance. As indicated in Table 1, 18 parents completed RCADS-P25 in sessions one and eight. Quantitative data also included evaluation ratings of CDK by 17 parents, see Supplementary Materials File S2 for questions.

#### 2.4.2. Qualitative

In addition to quantitative measures, we analysed the perspectives and viewpoints shared by parents regarding their experiences with CDK to strengthen our findings. Thematic analysis guided our interpretation and constructions of qualitative data to identify critical reflections and to create a multi-perspective view of participants’ CDK experiences [29]. Interview transcripts and evaluation data were coded line-by-line in NVivo12 software by the first author and reviewed at weekly meetings by the last author. We followed Braun and Clarke’s [30] six-phase process in our coding procedure, which involved familiarising ourselves with the data, creating initial codes, identifying themes, defining and naming the themes, and writing a report. While these phases suggest a linear progression, our analysis incorporated a flexible approach, allowing us to move back and forth between the phases as necessary. Strategies to address research rigour included a detailed audit trail, reflexive field notes, a team approach to analysis, and prolonged engagement with the subject matter.

## 3. Results

This study aimed to determine CDK’s acceptability by parents, its feasibility—its viability and practicality—and its effectiveness.

### 3.1. Quantitative

The RCADS-P25 scores indicate that the program is an effective intervention to support children’s mental health as it relates to anxiety. Analysis of the RCADS-P25 scores indicates a significant reduction in anxiety. RCADS-P25 results included a baseline (M/SD) of 15.2 (5.3) and a post-intervention (M/SD) of 10.3 (2.9), resulting in *p*-values that were <0.001. The findings revealed that the program positively and significantly impacted parental perceptions of their child’s anxiety. In addition, anonymous parental evaluation data indicated a high level of the program’s acceptability, as shown in Table 2.

The above quantitative results signal that CDK’s design, processes, and intervention activities were appropriate from the perspective of the parent. Consistent attendance rates and evaluation scores suggest that CDK is also an acceptable and feasible intervention to support children’s anxiety. These quantitative findings show promise. However, within the qualitative results, we find nuanced and rich stories to further support CDK as an effective, feasible, and acceptable intervention.

### 3.2. Qualitative

Following these quantitative results, qualitative data, including observational notes, participant textual evaluations, and interview data, were thematically analysed. After repeated engagement with the data, our findings were organised into four central themes: connections, a safe space, creative activities to make mistakes, and changes in mood over time. Direct quotes and written statements support our findings to illustrate themes through personal and social contexts.

#### 3.2.1. New Connections with Their Child

This theme highlighted how CDK encouraged opportunities to forge new bonds between parent and child, whether through the activities, the gallery space, or time spent together. For instance, parents commented,

*LOVE that I’m able to do this with my child—there are not many programs you can do with your child in general. Doing it with my daughter, learning about art and then being creative without limitation or an objective or outcome defined was what I liked most about the program.*

(A14, adult anonymous written feedback)

*The program has been an amazing reset for my child and I. The process of absorbing and creating art each Saturday has brought calm and joy. We feel so lucky to have been here. There was no pressure to create anything spectacular. This has been truly wonderful. For two hours a week the rest of the world has ceased to exist for me.*

(A3, adult anonymous written feedback)

*Great chance to take a break from a busy life and slow down. My child found a new outlet to be in the moment. We enjoyed the shared experience together. Great for my child but also for me. It’s been a long time since I have been creative, and great my child could access his own creativity.*

(A1, adult anonymous written feedback)

Another parent spoke about the way the space enabled parent–child connection:

*I think one thing about it is that it’s because we’re sharing this space, I feel like there’s a different connection between us. Like we’re both doing this thing where we come and have this experience and have this space and then we do the art part together. And so, I feel like it’s a bit of a bond between us.*

(P7, mother, interview feedback)

Connection through increasing topics of conversation during the challenging “tween” years (in between a child and a teenager, about 10–12) was evidenced in the following quote:

*The “tween” [years] is an age where it’s actually kind of hard to find a conversation point with your child that everybody’s comfortable with. And [CDK] is a great starting point. You’ve got a topic you can move on from it and it can lead to other things as well.*

(P4, mother, interview feedback)

The connectedness theme highlights the importance of a program that safely creates a space and time to share activities, experiences, and conversations to increase parent–child bonds.

#### 3.2.2. Creation of a Safe Space

The gallery setting has been established as a safe place to facilitate personal and community wellbeing [15]. The parents in our study articulated that the gallery and CDK “created a safe space for the children and parents to reflect and feel comfortable within this space”. One parent (P14) remarked, “being with her, in this space, knowing that we can come here together on Saturday and not have anything else to worry about”. These comments speak to the importance of an alternative neutral space that is not currently associated with anxiety, by either parent or child.

In addition, CDK took place in a metropolitan city. With this in mind, a parent (P9) spoke of how “coming to an appointment in the city has always been a lot of questions and anxiety around what we’re doing, where we’re going, holding my hand tightly” but that while her daughter “still doesn’t enjoy the city crowds and the pressure, [CDK] is a very safe space and she’s more than happy to come”.

#### 3.2.3. Open-Ended Creative Activities to Make Mistakes

This third theme, empowering children to make mistakes through open-ended creative activities is reflected in the following comments:

*I think is really important that she loves art, but she stopped painting because she was overthinking things, she didn’t think it was good enough, so this is brought back her creative side I really happy for it.*

(P14, mother, interview feedback)

*Art is a medium that has no right and wrong. You can’t do mistakes and that’s something kids with anxiety need.*

(A9, adult anonymous written feedback)

*He wants to excel and if he doesn’t excel, he’ll just take a step back and this [CDK] has encouraged him so much…this is also taught him the fact that you don’t have to excel in everything you do.*

(P13, mother, interview feedback)

CDK art activities were intentionally open-ended, not prescriptive, to encourage self-expression and creativity. Artworks could be finished in the time allotted or taken home. Some group art activities took place over weeks, to be added to as and when participants felt like it on the day.

#### 3.2.4. Positive Changes in the Child over Time

This last theme describes how parents reported a change in their child as a result of participating in CDK over the eight weeks. Parent P2 remarked that their child “always seems to come out in a good mood, always comes out of here happy, they get into a happy creative space, and that seems to last the day”. Similarly, parent P4 found “it’s a positive activity and it sets the mood for the whole day”. Parent P5 referenced that “there’s been a big jump in just him being happier and more confident, I think that’s the biggest takeaway [from CDK]”. Another parent said,

*So far, [CDK] really helped his confidence most of all. Yeah, I have noticed a change. It might be because of this program—I couldn’t correlate it with anything else other than this program. Yeah, it has definitely helped.*

(P11, mother, interview feedback)

*It’s too early to tell if it’s made a difference to my child’s unique issues, being alone, being in the dark, but I can see a little bit of difference. I can now drop him on the corner to run that last bit on his own that before he wouldn’t do.*

(P15, mother, interview feedback)

Comments from the parents signify that the time spent in the eight-week program created a change in their child that endured beyond the program, including positive changes to their child’s happiness and self-confidence.

## 4. Discussion

This study aims to understand CDK’s effectiveness, acceptability, and feasibility (viability and practicality) with children with anxiety through their parents’ perspectives and feedback. Our inclusion of quantitative and qualitative analysis increases the robustness of determining whether CDK is a practical, viable, effective, and acceptable mental health support for children with anxiety.

The quantitative parental RCADS results indicate an improvement in their child’s anxiety levels as a direct outcome of the project, signalling its effectiveness. In addition, when examining the evaluation form results, all parents view CDK as a resource they would recommend to another parent to support a child who experiences anxiety. These two findings, taken together, indicate a high level of CDK’s effectiveness, acceptability, and feasibility by parents.

Notwithstanding these quantitative results, a more nuanced understanding of CDK’s impact on the child and parent can be understood through qualitative enquiry. This approach embraces the complexity of human health and social behaviours to enhance research findings [26,27]. Our findings, organised through people–place–activity–time themes, mirror the approach to creating CDK. The interaction of CDK components—who takes part, where it is located, what takes place and how, over eight sessions—build upon each other to strengthen the program and results.

The program facilitates a safe place and time for parents and children to be reflective and creative together to broaden and deepen parent–child bonds. CDK creates extended moments of calm to connect parent and child, providing parents with opportunities to explore their children and themselves in new ways to foster understanding, echoing Stock et al.’s [21] findings. Galleries are social institutions that promote personal growth and wellbeing through a deeper sense of the self and one’s surroundings, circumventing the stigmatisation of a clinical or therapy setting [15,23]. Situating CDK in a gallery creates a calming, reflexive space to increase the sharing of ideas, activities, experiences, and conversations to increase parent–child connectedness.

Our findings illustrate depictions of improved mood, confidence, and sense of empowerment in the child, qualities associated with resilience and mental wellbeing [14,20]. Open-ended activities provide opportunities for connection, creativity, and experimentation—sources of strength for improving mental health [20]. Comments from the parents signify that CDK creates a change in mood and attitude that lasted beyond the immediacy of the program. Indeed, one mother emailed us a month after the program ended, writing the following about her son’s school report: “two words which were phenomenal to see printed! These words were ‘resiliency’ and ‘confidence’! Two such attributes that have never been associated with [him] and I am almost certain that [CDK] was the main instigator of these”.

Early interventions can improve young people’s lifelong mental health and wellbeing [5]. By engaging both parent and child in this inclusive, non-stigmatising arts-based mental health intervention, a safe and creative community approach to support wellbeing occurred for child and parent. Our results reinforce Mak and Fancourt’s [19] findings that the positive impact of AEPs may be magnified if parents are involved in arts activities with their children. Our findings reflect the complex interactions of people–place–activity–time in the program. By this, we mean that CDK takes place in a peer group setting in a calming, meditative space (gallery) with reflective and creative activities that encourage story sharing over an extended period to build connections to inner thoughts, feelings, family members, peers, the gallery, artworks, and creativity. The program’s components interact and build off each other to produce positive mental health outcomes for participants.

### Limitations

As this study was a pilot program, our findings were limited to a small group of urban children and families. Participation in CDK was established by parental belief that their child experienced anxiety (RCADS were not used to diagnose). Three-month RCADS follow-up had a low response rate, which, in future studies, we will incentivise with art gift packs. Furthermore, given the focus on supporting child anxiety, the authentic voice of children is absent in the paper. Instead, this paper’s findings are derived chiefly from mothers’ perspectives. In addition, while many parents responded that CDK was a great “reset” for them and their children, a quantitative measure of parental wellbeing before and after the program is missing.

In future CDK programs, we plan to tap into children’s experiences in more creative ways, befitting the ethos of this study. We will also quantitatively measure parents’ wellbeing levels to understand the impact of CDK on them. We acknowledge the limitations of the current research, thereby warranting further studies with a control group and improved longer-term follow-up to establish reliable causality and longitudinal findings.

## 5. Conclusions

This study reveals that CDK positively and significantly impacted the parents’ reported anxiety level of their child, indicating its acceptability as a mental health support. Our qualitative and quantitative findings illustrate the role CDK can offer as a practical, viable, effective, and acceptable mental health support for children with anxiety. The program fosters increased parent–child connections as a significant outcome, facilitated by the interplay of extended time, reflexive activities, and space. In addition, over eight weeks, children are empowered through open-ended creative activities to connect to their feelings, thoughts, and imagination to build self-confidence and resilience while challenging the need for perfectionism. Comments from the parents signify that CDK creates positive mental health outcomes that last beyond the immediacy of the program.

This study adds to the small but growing evidence base supporting the role of community and cultural care in youth mental health and wellbeing. Health professionals, decision-makers and policymakers can look outside traditional care systems to improve community health and mental wellbeing. Specifically, they can look to community programs, like CDK, that emphasise self-expression, creativity, and enjoyment to nurture positivity and wellbeing. Moreover, public galleries and cultural organisations can use the findings from this study to further develop mental health and wellbeing programs as a strategic and integral part of their work. Our results support the limited positive evidence about arts engagement programs, children, and mental health, contributing to yet highlighting the need for further research in this age group.

## Figures and Tables

**Table 1:** Data collection by type.

Data Collection Type	Number of Participants	Methodology	Session Administered
Anonymous evaluation forms	17 ^1^	Qual and Quant	8
Oral interviews	19	Qualitative	8
Observational notes	1 (first author)	Qualitative	1–8
RCADS—P25	18	Quantitative	1, 8 (+3 mos.)

^1^ Two parents filled in one evaluation form for two children.

**Table 2:** Parental evaluation results.

Question	Mean Score/Maximal Score	No. of Responses
Would recommend it to other parents	9.9/10	17
Overall satisfaction	9.8/10	17
Met their needs	4.8/5	17
Enjoyed participating	4.7/5	17
Distracted from stressors	4.4/5	17
Encouraged meaningful participation	4.6/5	17
Encouraged social connectedness	4.4/5	17

## Data Availability

Data are unavailable due to the condition of ethical approval.

## Supplementary Materials

The following supporting information can be downloaded at: https://www.mdpi.com/article/10.3390/ijerph20186771/s1, File SI: Culture Dose for Kids; File S2: Culture Dose for Kids Completion Satisfaction Questionnaire—Parent.
