# Supplementary material for: Parents’ Perspectives of an Arts Engagement Program Supporting Children with Anxiety
Source: Int J Environ Res Public Health. 2023 Sep 16;20(18):6771. doi: 10.3390/ijerph20186771 (PMC10531464; doi:10.3390/ijerph20186771)
Supplement: Supplementary file 1 [file ijerph-20-06771-s001.zip › File S2 Culture Dose for Kids Completion Satisfaction Questionnaire - Parent.pdf]

Black Dog Institute

Today's Date: 27 Nov 2022 TRG

Culture Dose for Kids Completion Satisfaction Questionnaire - Parent

Questions	0	1	2	3	4	5	6	7	8	9	10	Please comment – what influenced your decision to rate this way?
1. Based on your experience with the Culture Dose for Kids Program, how likely would you be to recommend the program to someone in your position? Please rate on a scale of 0 to 10, where 0 is not at all likely, and 10 is extremely likely.												
2. On a scale of 0 to 10, where 0 is the least and 10 is the most satisfied, how would you rate your overall satisfaction with the program?												

Questions	Not at all	Slightly	Somewhat	Moderately	Extremely	Comments
3. Did you feel the program met your child's individual needs and catered to their creative strengths, talents / weaknesses?	1	2	3	4	5	
4. Did you enjoy participating in the program?	1	2	3	4	5	
5. Did the program have any impact on you and your child?	1	2	3	4	5	
6. Did the program encourage you to identify and participate in creative activities that are meaningful to you and your child?	1	2	3	4	5	
7. Did the program support you to improve your social connectedness with your child and others?	1	2	3	4	5	

Today's Date: 27 Nov 2022 TRG

Black Dog Institute

8. What did you like most about the program?

____

____

____

9. Is there anything you would change about the program / How could the program be improved?

☐ Yes ☐ No

If YES, please provide details:

____

____

OPTIONAL: Please feel to add any additional comments here.

Thank you for your feedback 😊
