# Supplementary material for: Parents’ Perspectives of an Arts Engagement Program Supporting Children with Anxiety
Source: Int J Environ Res Public Health. 2023 Sep 16;20(18):6771. doi: 10.3390/ijerph20186771 (PMC10531464; doi:10.3390/ijerph20186771)
Supplement: Supplementary file 1 [file ijerph-20-06771-s001.zip › File SI Culture Dose for Kids.pdf]

Black Dog Institute

Culture Dose for Kids

At the completion of the 8-week arts engagement program, participants will be asked to discuss their experiences with the program and to explore how engagement in creative activities can support mental wellbeing, social inclusion and resiliency. Interviews will be run by the peer researcher and held at the same time and place as the art program.

Participants will be reminded that what they say will have no impact on their relationship with UNSW, The Black Dog Institute or the Art Gallery of NSW.

The focus group will be digitally voice recorded, as per the participant consent form.

Interview Guideline Questions

1. Please describe what you most enjoyed about the arts engagement program, and why.
2. Can you describe what you least enjoyed, and/or any barriers you experienced to participate in the arts engagement program?
3. What do you feel you got out of the arts engagement program e.g. new skills, knowledge, interests, friendships, connections, other?
4. Based on your observations, how do you think it impacted your child?
5. How do you intend to maintain or continue engaging in creative activities with your child in the future?
6. Based on your experiences with the Culture Dose for Kids, how do you think creative community activities, such as the Culture Dose for Kids, can help other kids with anxiety/parents of kids with anxiety?
7. Is there anything you would change about the program, for creative activities to better meet the wellbeing and social inclusion needs of kids with anxiety? Why? How?

Thank participants for their time and commitment to the arts engagement program and the research study.
